# Monoclonal antibody 10A5 recognizes an antigen unique to the water-insoluble 25/45 membrane fraction of the rat ocular lens

**DOI:** 10.1186/2193-1801-2-500

**Published:** 2013-10-02

**Authors:** Joseph K Whitman, Abigail F Alviar, Charles R Fleschner, Melissa K Stuart

**Affiliations:** Northeast Regional Medical Center, Otolaryngology/Facial Plastic Surgery Residency Program, 315 S Osteopathy Ave, Kirksville, MO 63501 USA; Phoenix Children’s Hospital/Maricopa Medical Center Pediatric Residency Program, 1919 E Thomas Rd, Phoenix, AZ 85016 USA; Department of Biochemistry, A T Still University, Kirksville College of Osteopathic Medicine, 800 W Jefferson St, Kirksville, MO 63501 USA; Department of Microbiology/Immunology, A T Still University, Kirksville College of Osteopathic Medicine, 800 W Jefferson St, Kirksville, MO 63501 USA

**Keywords:** Ocular lens, Plasma membrane domains, Monoclonal antibodies, Gangliosides

## Abstract

The water-insoluble 25/45 fraction and non-sedimenting membrane fraction (NSMF) are two membrane preparations isolated from the ocular lens. The fractions are postulated to represent distinct subdomains of the lens with unique functions. However, attempts to distinguish between the two fractions by detecting proteins present in one fraction but absent from other have been unsuccessful. In this study, we exploited the ability of the mouse immune system to detect antigenic differences between the 25/45 fraction and NSMF isolated from the lenses of 20-day-old rats. We generated a monoclonal antibody (MAb 10A5) that reacts with a ganglioside-like antigen that is present in the 25/45 fraction but absent from the NSMF. Restriction of the antigen to the 25/45 fraction in 20-day-old animals supports the hypothesis that the 25/45 fraction and NSMF represent different subdomains within the ocular lens.

**Electronic supplementary material:**

The online version of this article (doi:10.1186/2193-1801-2-500) contains supplementary material, which is available to authorized users.

## Background

The lens fiber cell plasma membrane is organized into subdomains of clustered macromolecules that differ in composition from the majority of the bilayer (Raguz et al. 2008). Such domains include adhesive structures (cellular synapses, substrate adhesions, and fiber cell junctions), membrane invaginations (clathrin-coated pits and caveolae), and less well-defined domains such as cholesterol-rich lipid rafts and lectin-glycoprotein lattices (Lajoie et al. 2009). The varied composition of the domains facilitate unique functions. Lens fiber junctions, which contain proteins called connexins, maintain homeostasis within the fiber cells by facilitating the transfer of water, ions, and low molecular weight compounds between adjacent, communicating cells (Fleschner and Cenedella 1991). Caveolae play roles in lipid transport, endocytosis, signal transduction, and cell transformation (Perdue and Yan 2006). Cholesterol crystalline domains are essential for maintaining lens transparency (Borchman et al. 1996; Jacob et al. 1999,2001), most likely by interfering with cataractogenic aggregation of α-crystallin at the membrane surface (Tang et al. 1998).

In a previous study, Fleschner and Cenedella (1993) described the isolation of a non-sedimenting membrane fraction (NSMF) from the water-soluble fraction (WSF) of bovine lens. The NSMF differed in several regards from the water-insoluble sedimenting membrane fraction (SMF): the NSMF contained fewer fiber junction structures, a greater amount of total lipid relative to total membrane protein, and less cholesterol relative to phospholipid than the SMF. In a follow-up study, it was shown that the NSMF contained a greater concentration of triacylglycerol than the SMF, and that there was an inverse relationship between membrane cholesterol and triacylglycerol content (Fleschner and Cenedella 1997). It was suggested that triacylglycerol-rich domains might exist as oily pools to allow diffusion of lipophilic molecules, thus providing a transport mechanism across fiber cell plasma membranes with diminished transport activities (May et al. 1986; Fleschner and Cenedella 1993).

More recently, an additional lens membrane preparation that we call the "25/45 fraction" was isolated. Like the SMF, the 25/45 fraction is hypothesized to be distinct from the NSMF. The 25/45 fraction is prepared by homogenizing lenses in aqueous buffer without chaotropic agents, sedimenting the water-insoluble fraction, and then subjecting the water-insoluble fraction to ultracentrifugation through a discontinuous sucrose density gradient (Fleschner 1998). The 25/45 fraction is so-called because it is isolated from the interface between 25% and 45% sucrose. The 25/45 fraction contains the full complement of extrinsic (8 M urea-soluble) proteins found in the lens "native" plasma membrane in vivo (Cenedella and Fleschner 1992). Crystallins account for approximately 90% of the extrinsic protein, with the remainder comprising cytoskeletal and other proteins (Cenedella and Fleschner 1992). A comparison of the cytoskeletal components vimentin, phakinin, and filensin in the NSMF and 25/45 fractions showed that these proteins differed quantitatively but not qualitatively between the two membrane fractions in both bovine and rat lenses (Fleschner 1998, 2002).

Because prior studies did not reveal proteins uniquely associated with the 25/45 fraction or NSMF, we undertook the current investigation to determine whether antigenic differences could be detected using monoclonal antibodies raised separately to the 25/45 fraction and NSMF isolated from 20-day-old rats. Our goal was to reveal additional differences between the two membrane fractions to support the hypothesis that the 25/45 fraction and NSMF represent distinct lens subdomains. Here we describe the production of a monoclonal antibody (MAb 10A5) specific for an antigen that in 20-day-old rats is restricted to the 25/45 fraction. The antigen appears to be biochemically related to the gangliosides.

## Results

### Screening of hybridoma supernatants by ELISA and immunoblotting

**Figure 1:**
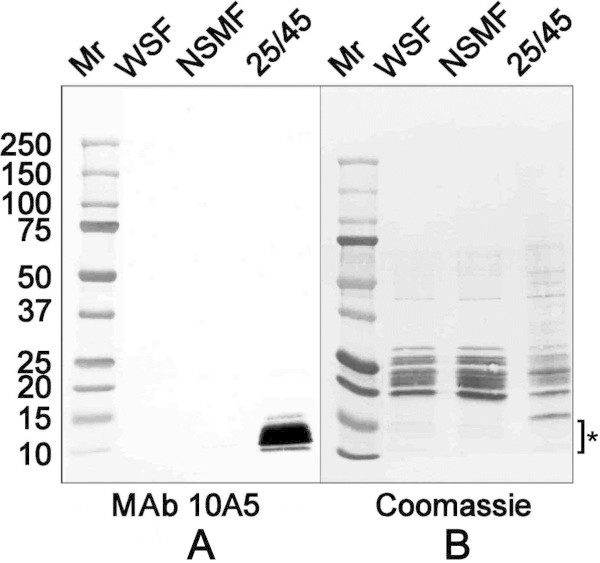
**Immunoblot analysis of lens membrane fractions isolated from 20-day-old rats. (A)** Lens membrane fractions separated by electrophoresis through 5-20% gradient gels were transferred to PVDF and immunoblotted with MAb 10A5. WSF: water soluble fraction, 10 μg. NSMF: non-sedimenting membrane fraction, 5 μg. 25/45 fraction, 5 μg. **(B)** A duplicate gel was stained with Coomassie Blue R-250. Note that the 10–15 kD antigen reactive with MAb 10A5 did not stain with Coomassie Blue (area marked by the asterisk), although numerous proteins outside that mass range took up the dye. Molecular weight standards (Mr) are Bio-Rad Precision Plus dual color markers (5 μl) and are expressed in kD.

**Figure 2:**
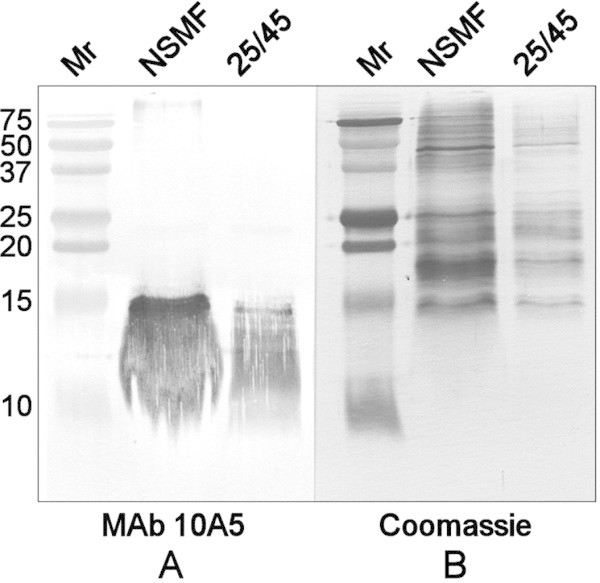
**Immunoblot analysis of lens membrane fractions isolated from 75-day-old rats. (A)** Lens membrane fractions separated by electrophoresis through 18% polyacrylamide gels were transferred to PVDF and immunoblotted with MAb 10A5. The NSMF and 25/45 fraction were applied at 5 μg/lane. **(B)** A duplicate gel was stained with Coomassie Blue R-250. Mr, Bio-Rad Precision Plus dual color markers (5 μl).

Approximately 1600 hybridoma supernatants were tested by indirect ELISA against the 25/45 fraction and NSMF isolated from 20-day-old rats. Of these, nearly 10% recognized the homologous membrane fraction used for mouse immunization. All but one of the supernatants (MAb 10A5) also reacted with the heterologous membrane fraction. As shown in Figure 1A, immunoblotting revealed that MAb 10A5 recognized a low molecular weight antigen that was unique to the 25/45 fraction, but absent from the water-soluble supernatant fraction (WSF) and the NSMF isolated from 20-day-old rats. The antigen migrated as a series of bands between 10 kD and 15 kD and could be visualized by immunoblotting (Figure 1A), but not by staining with Coomassie Blue R-250 (Figure 1B). In blots of lens fractions prepared from 75-day-old rats (Figure 2A), the antigen was detected in both the NSMF and 25/45 fraction. The multi-band pattern in samples from 75-day-old animals was less distinct than that seen in 20-day-old animals.

### Enzymatic deglycosylation of the 25/45 fraction

**Figure 3:**
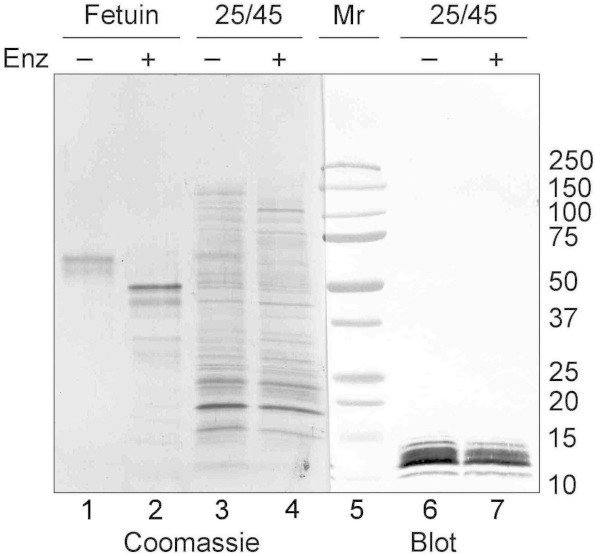
**Enzymatic deglycosylation of the 25/45 fraction isolated from 20-day-old rats.** The control glycoprotein bovine fetuin (1 μg/lane) and the 25/45 fraction (8 μg/lane) were incubated with (+) or without (-) deglycosylation enzymes (Enz) prior to their electrophoresis through a 5-20% gradient gel and transfer to PVDF. Deglycosylation of fetuin resulted in a major shift in its molecular weight (compare lanes 1 and 2). Minor differences in Coomassie Blue staining patterns are evident between the untreated (lane 3) and deglycosylated (lane 4) 25/45 fraction samples. However, deglycosylation of the 25/45 fraction failed to alter the electrophoretic mobility or immunoreactivity of the antigen recognized by MAb 10A5 (compare lanes 6 and 7).

The 25/45 fraction from 20-day-old rats was evaluated for carbohydrate residues using an enzymatic deglycosylation procedure that removes N-linked and O-linked carbohydrates from glycoproteins (Figure 3). The presence of glycoproteins in the 25/45 fraction was demonstrated by the minor differences observed between untreated (lane 3) and deglycosylated (lane 4) samples stained with Coomassie Blue R-250. These differences were most apparent between 50 and 150 kD. Deglycosylation of the 25/45 fraction did not change the electrophoretic mobility of the antigen reactive with MAb 10A5 or the intensity of its recognition by the antibody (lane 7) compared to the untreated control (lane 6). Deglycosylation of the bovine fetuin positive control (lane 2) resulted in a major shift in its molecular weight compared to the untreated control (lane 1).

### Immunoprecipitation of the antigen reactive with MAb 10A5, followed by 1-D and 2-D electrophoresis

**Figure 4:**
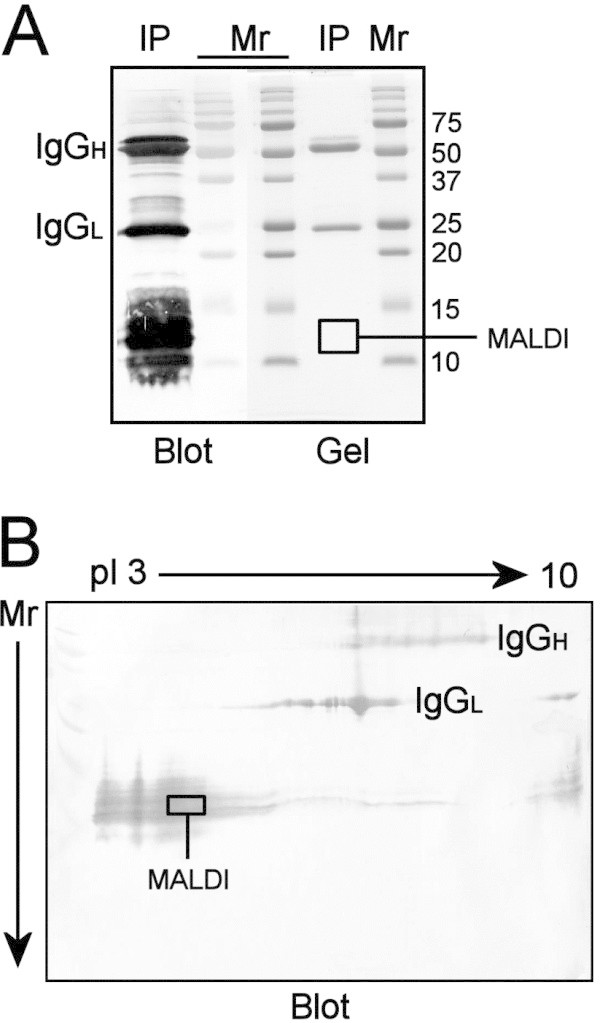
**One-dimensional (1-D) and two-dimensional (2-D) electrophoresis of the antigen immunoprecipitated by MAb 10A5.** The antigen reactive with MAb 10A5 was concentrated by immunoprecipitation from the 25/45 fraction of 20-day-old rats using MAb 10A5-coated magnetic beads. The IgG heavy (IgG_H_) and light (IgG_L_) chains comprising MAb 10A5 are marked. **(A)** After 1-D electrophoresis, the immunoprecipitate (IP) was immunoblotted with MAb 10A5 (Blot) or stained with colloidal Coomassie Blue G-250 (Gel). The area of the gel submitted for MALDI-ToF/MS is indicated by the box. **(B)** After 2-D electrophoresis, the immunoprecipitate was immunoblotted with MAb 10A5. The boxed area was excised from replicate 2-D gels stained with colloidal Coomassie Blue G-250, and the gel slices were analyzed by MALDI-ToF/MS. pI, isoelectric focusing point.

The antigen reactive with MAb 10A5 was concentrated by immunoprecipitation from the 25/45 fraction isolated from 20-day-old rats. The immunoprecipitate was subjected to one-dimensional (1-D) (Figure 4A) and two-dimensional (2-D) electrophoresis (Figure 4B) prior to analysis by matrix-assisted laser desorption/ionization-time-of-flight/mass spectrometry (MALDI-ToF/MS). The IgG heavy (IgG_H_) and light (IgG_L_) chains comprising MAb 10A5 were evident at 55 kD and 25 kD, respectively, in both 1-D and 2-D gels. In the blot of the 1-D gel (Figure 4A), the immunoprecipitated antigen was strongly recognized by MAb 10A5 within the 10–15 kD mass range, but the antigen remained unstained by colloidal Coomassie Blue G-250 in the replicate gel. None of the additional stains, including Alcian blue, silver nitrate, imidazole-zinc sulfate, Stains-all, and Stains-all counterstained by silver nitrate, stained the antigen even after it was concentrated by immunoprecipitation (data not shown). In blots of 2-D gels (Figure 4B), the antigen focused toward the anode and within the expected 10–15 kD mass range. Areas of the 1-D and 2-D gels submitted for MALDI-ToF/MS are marked by boxes in Figure 4A and 4B.

### MALDI-ToF/MS

Proteins in gel slices containing the antigen recognized by MAb 10A5 were provisionally identified by MALDI-ToF/MS. The mass lists compiled for the 1-D versus 2-D gel slices were completely unique, with no proteins identified in common. The sole protein identified with confidence in the 1-D gel slice was structural maintenance of chromosomes 1-like 1 (SMC1, gi 13928946), a protein involved in sister chromatid cohesion during the mitotic cell cycle (Zou 2011). An Excel file containing the peptide summary report for the 1-D gel slice has been provided [see Additional file 1]. The sole protein identified with confidence in gel slices excised from duplicate 2-D gels was anionic trypsin-1 precursor (gi 6981420), which may have been a contaminant contributed by the trypsin preparation used in the MALDI-ToF procedure. Details of the peptide summary report for the 2-D gel slices are presented as an additional Excel file [see Additional file 2].

### Protease digestion of the 25/45 fraction from 20-day-old rats

**Figure 5:**
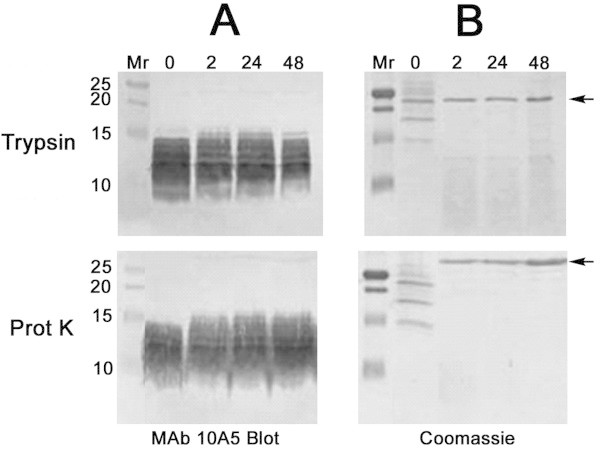
**Protease resistance of the antigen recognized by MAb 10A5.** Samples of the 25/45 fraction (20 μg) removed after 0, 2, 24, and 48 hours of digestion with trypsin or proteinase K (Prot K) were run on duplicate 18% resolving gels (10 μg/lane). **(A)** Digests from one gel were probed with MAb 10A5 by immunoblot analysis. **(B)** The duplicate gel was stained with Coomassie Blue R-250. Arrows mark trypsin and proteinase K at 23.8 kD and 28.9 kD, respectively.

Trypsin or proteinase K digestion of the 25/45 fraction for up to 48 h had no effect on the immunoreactivity or electrophoretic mobility of the 10–15 kD antigen reactive with MAb 10A5 (Figure 5A). In contrast, membrane proteins in the 25/45 fraction were completely destroyed by protease treatment, as demonstrated by Coomassie staining of the digests after SDS-PAGE (Figure 5B). Within 2 h of digestion, the only discernible proteins that remained in the digests were the proteases trypsin and proteinase K, which migrated to 23.8 kD and 28.9 kD, respectively (arrows, Figure 5B).

### Folch extraction, thin layer chromatography, and dot blot immunoassay

**Figure 6:**
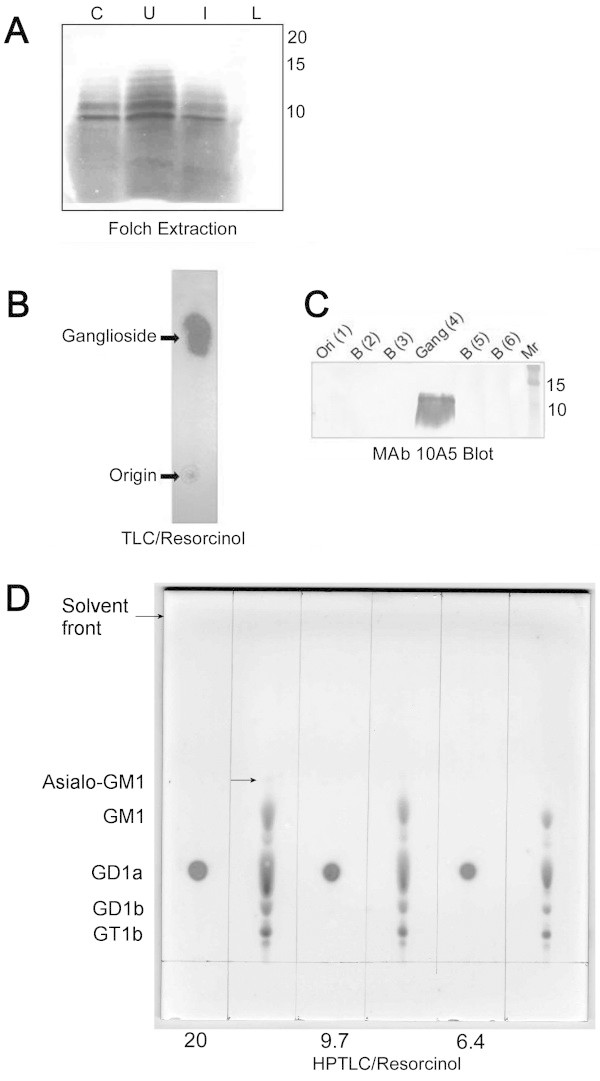
**Folch extraction and thin-layer chromatography of the antigen unique to the 25/45 fraction. (A)** Fifteen-μl aliquots of the upper phase (U), lower phase (L), and interface (I) obtained by Folch extraction of the 25/45 fraction isolated from 20 day-old rats were separated by SDS-PAGE through 18% resolving gels and probed with MAb 10A5. Unextracted 25/45 fraction (5 μg) was included as a positive control (C). **(B)** Upper phase obtained by Folch extraction from 200 μg of 25/45 fraction protein was subjected to TLC through 20 × 20 cm silica gel H plates and stained with resorcinol to visualize gangliosides. **(C)** Silica gel bands scraped from a replicate TLC plate were eluted and subjected to SDS-PAGE through 18% resolving gels. After transfer to PVDF, the membrane was immunoblotted with MAb 10A5. Note that immunoreactivity was restricted to the area of the plate containing the ganglioside (Gang). No reactivity was noted at the origin (Ori) or in other bands (B) scraped from the lane containing the upper phase. **(D)** Ganglioside extracted by the Folch method from the equivalent of 20, 9.7, and 6.4 μg of 25/45 fraction protein was subjected to high performance TLC (HPTLC) through 10 × 10 cm silica gel 60 plates and stained with resorcinol. Bovine mixed ganglioside standards were applied at 18.8, 9.4, and 4.7 μg (per ganglioside) in alternating lanes. The arrow marks the asialo-GM1 ganglioside.

**Figure 7:**
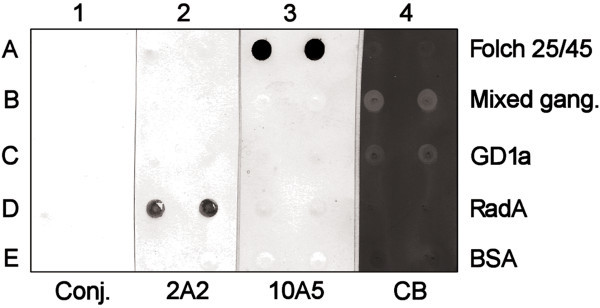
**Ganglioside dot blot immunoassay employing MAb 10A5.** Rows of a PVDF membrane were spotted with: **(A)** the Folch upper phase extracted from 2 μg of 25/45 fraction protein, **(B)** 5 μg of bovine mixed ganglioside standards, **(C)** 1 μg of purified bovine GD1a, **(D)** 50 ng of the negative control protein RadA-6xHis, and **(E)** 2.5% BSA in 50% methanol. The antigens were probed with: (1) alkaline phosphatase-conjugated secondary antibody in the absence of primary antibody; (2) MAb 2A2, an isotype-matched negative control antibody specific for the irrelevant bacterial protein RadA-6xHis; and (3) MAb 10A5. Panel 4 was stained with Coomassie Blue R-250 and destained in 40% methanol/10% acetic acid to reveal white ganglioside spots against a blue background.

**Figure 8:**
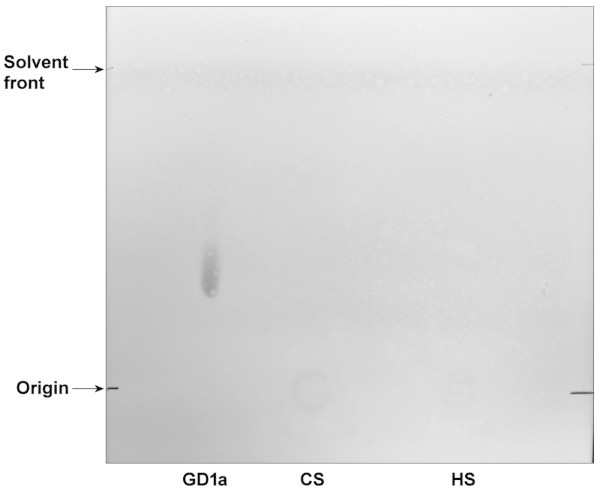
**HPTLC of proteoglycans.** The proteoglycans chondroitin sulfate (CS) and heparan sulfate (HS) were applied at 10 μg/spot to an HPTLC silica gel 60 plate. The plate was developed in chloroform:methanol:0.2%CaCl_2_::55:45:10 and stained with resorcinol as described for the plate shown in Figure 6D. The proteoglycans failed to migrate from the origin and stained poorly with resorcinol, in contrast to the GD1a ganglioside standard and with the antigen recognized by MAb 10A5.

Folch extraction of the 25/45 fraction isolated from 20-day-old rats yielded three distinct phases: an upper phase, a flocculent interface, and a lower phase. Both the upper phase and interface were reactive with MAb 10A5 in immunoblots (Figure 6A). Thin-layer chromatography of the upper phase in a solvent system comprising propanol/water (7:3 v/v) resolved a single spot that stained with resorcinol (Figure 6B). When eluates from silica gel bands scraped from a duplicate TLC plate were probed with MAb 10A5 by immunoblotting, MAb 10A5 reactivity was present only in the band that corresponded with the ganglioside spot revealed by resorcinol staining (Figure 6C). Resolution of the immunoreactive spot through HPTLC plates in a solvent system comprising chloroform/methanol/0.2% aqueous CaCl_2_ (55:45:10 v/v/v) demonstrated that it co-migrated with the GD1a bovine ganglioside standard (Figure 6D). However, neither purified bovine GD1a nor any of the additional bovine brain gangliosides in the standard mixture reacted with MAb 10A5 in a dot blot immunoassay (Figure 7, panel 3). The proteoglycans chondroitin sulfate and heparan sulfate failed to migrate from the application origin on HPTLC plates developed with the chloroform/methanol/0.2% aqueous CaCl_2_ (55:45:10 v/v/v) solvent system and stained poorly with resorcinol (Figure 8).

## Discussion

In this study, we demonstrated that the 25/45 membrane fraction differs antigenically from the NSMF isolated from the ocular lenses of 20-day-old rats. We generated a monoclonal antibody, MAb 10A5, specific for a ganglioside-like antigen unique to the 25/45 fraction that was absent from the NSMF in 20-day-old animals. The presence of the antigen in one membrane fraction, but not the other, supports the hypothesis that the 25/45 fraction and NSMF represent distinct subdomains within the ocular lens.

We initially assumed that the antigen recognized by MAb 10A5 is a protein or modified protein based on its electrophoretic mobility through polyacrylamide gels. However, an extensive battery of tests performed on the antigen failed to reveal any evidence of a proteinaceous component. In the first series of tests, the antigen was concentrated by immunoprecipitation from the 25/45 fraction and subjected to SDS-PAGE. Gels containing the antigen were incubated in routine and specialized stains. Neither silver stain (estimated sensitivity, 5–10 ng per protein band) nor colloidal Coomassie Blue G-250 (sensitivity, ~30 ng/band) detected the antigen. The cationic dye Stains-all (Goldberg and Warner 1997), which stains sialoglycoproteins and phosphoproteins blue, proteoglycans purple, and less acidic proteins pink, failed to detect the antigen. Stains-all counterstained with silver nitrate, a combination that enables detection of sub-nanogram quantities of phosphoproteins (Goldberg and Warner 1997), did not stain the antigen. The proteoglycan stain Alcian blue (Wall and Gyi 1988) and the reverse stain imidazole-zinc sulfate (Castellanos-Serra et al. 1999; Hardy et al. 1997), were also ineffective in antigen visualization. In each experiment, a replicate gel was immunoblotted with MAb 10A5 to ensure the presence of the antigen within the 10–15 kD mass range.

Next, the antigen was evaluated as a glycoprotein by subjecting the 25/45 fraction to enzymatic deglycosylation and examining immunoblots probed with MAb 10A5 for changes in antigen mass or intensity of antibody recognition. No changes in the immunoblot pattern were observed after deglycosylation, suggesting that the antigen reactive with MAb 10A5 is not a glycoprotein that contains N- or O-linked carbohydrates. We then attempted to identify the antigen by MALDI-ToF/MS. The antigen was concentrated and separated from other components in the 25/45 fraction by immunoprecipitation, followed by 1-D and 2-D electrophoresis. Gel slices were submitted for analysis after ensuring the antigen’s presence by clipping pieces from each end of the slices and probing the pieces with MAb 10A5. The peptide mass lists compiled for the 1-D and 2-D gel slices were completely different from one another with no proteins identified in common, despite irrefutable evidence of the antigen’s presence in all gel slices submitted for analysis. We interpreted the MALDI-ToF/MS results as compelling evidence that the antigen recognized by MAb 10A5 is not a protein. The proteins listed in the MALDI-ToF/MS reports were most likely contaminants, especially given that 90% of these proteins were present in such low quantity that they could not be identified with confidence.

To confirm our hypothesis that the antigen recognized by MAb 10A5 is not a protein, we incubated the 25/45 fraction with trypsin and proteinase K, and found that the protein digestion protocols had no effect on the antigen’s mass or immunoreactivity. Given these results, we considered other, non-protein classes of macromolecules in subsequent attempts to identify the antigen.

During extraction of the 25/45 fraction by the Folch method, the antigen reactive with MAb 10A5 partitioned to the upper phase, which is known to contain both proteins and gangliosides (Folch et al. 1957). Thin layer chromatography and resorcinol staining of the upper phase revealed the presence of a ganglioside-like spot that, when eluted from the TLC plate, proved highly immunoreactive with MAb 10A5. On high performance TLC plates developed with the chloroform/methanol/0.2% aqueous CaCl_2_ (55:45:10 v/v/v) solvent system that is well-suited for ganglioside separation (Schlosshauer et al. 1988), the immunoreactive spot migrated with the same mobility as the GD1a ganglioside standard. In a subsequent dot blot immunoassay, MAb 10A5 failed to recognize purified bovine GD1a or any of the additional gangliosides in the standard mixture, which included GT1b, GD1b, GM1, and asialo-GM1. Normal rat lenses have been shown to contain GM1, GD1a, GD1b, GT1a, GT1b, GQ1b, GM3, GD3, GT3, GT1c, GQ1c, and GP1c (Saito and Sugiyama 2000a, b). MAb 10A5 may recognize a lens-associated ganglioside, *e.g.* GD3 or GT3, whose mobility through TLC plates is similar to that of GD1a.

Because we are aware that proteoglycans can be resistant to proteases (Seldin et al. 1985), we considered the possibility that the antigen reactive with MAb 10A5 might be a proteoglycan that would be unaffected by incubation in trypsin or proteinase K. Therefore, we subjected the proteoglycans chondroitin sulfate and heparan sulfate to HPTLC in the chloroform/methanol/0.2% aqueous CaCl_2_ (55:45:10 v/v/v) solvent system and stained the chromatograms with resorcinol. In sharp contrast to the ganglioside-like antigen recognized by MAb 10A5, the proteoglycans failed to migrate from the application origin and stained very poorly with resorcinol. This result demonstrated that the antigen reactive with MAb 10A5 is biochemically distinct from the proteoglycans. The result also was consistent with the failure of Stains-All and Alcian blue to detect the antigen in polyacrylamide gels.

The multi-band pattern yielded by the ganglioside-like antigen in western blots probed with MAb 10A5 is intriguing. There is scant literature that describes how free gangliosides behave in SDS-PAGE gels. A report by Heuser et al. (1974) showed that purified gangliosides migrate behind the dye front when subjected to polyacrylamide gel electrophoresis in the presence of SDS. Gangliosides can self-associate and may interact with proteins through nonspecific hydrophobic interactions (Fukunaga et al. 2012; Osborne et al. 1982). We believe that the ganglioside-like antigen recognized by MAb 10A5 migrates to the area of the gel behind the dye front, where it interacts nonspecifically with protein fragments that are present in the same area. We further believe that this interaction explains why we see multiple antibody-reactive bands on Western blots of the 25/45 lens membrane preparation.

Gangliosides play an important role in the formation and stabilization of specific cell membrane lipid domains (Sonnino et al. 2007). Gangliosides are ceramide-derived glycolipids with one or more sialic acid residues attached via glucose to the primary hydroxyl group of the sphingosine backbone. The carbohydrate moiety always contains galactose in addition to glucose and sialic acid, and may also contain N-acetyl galactosamine. The hydrophobic ceramide contains a fatty acid and is inserted into the plasma membrane, with the hydrophilic oligosaccharide headgroups protruding into the extracellular medium (Cavallotti and Cerulli 2008; Sonnino et al. 2007). The amphiphilic character of the ganglioside manifests several physico-chemical properties that contribute to microdomain formation. These include lipid transition temperature, oligosaccharide headgroup geometries that favor ganglioside clustering and packing, the ability of the headgroups to interact with water molecules, and the capacity of the gangliosides to form hydrogen bonds at the lipid-water interface (Sonnino et al. 2007). Headgroup composition greatly influences the formation of positive and negative surface curvatures within the cell membrane. For example, in caveolae, gangliosides are distributed so that those with the highest surface area (*i.e.*, the largest oligosaccharide headgroups) are located on the edges of the invaginations where the surface has the greatest positive curvature, with cholesterol positioned in the inner part (Sonnino et al. 2007). Such an arrangement forms a microdomain of reduced membrane fluidity, where the components necessary to carry out such functions as receptor trafficking and signal transduction are held in close proximity to one another (Raguz et al. 2008; Sonnino et al. 2007).

Alterations in lens ganglioside composition may influence cataract formation. Early studies suggested that human cataractous lenses consist of a simple pattern of GM3 and GM1 (Sarkar and Cenedella 1982; Windeler and Feldman 1970), but later reports revealed a more complicated ganglioside pattern (Swindell et al. 1988; Tao and Lee 1986). In particular, Ogiso (1998) and Ogiso et al. (1990) showed that mature cataractous lenses contain an increased level of a slow-moving ganglioside when compared to immature cataractous lenses removed from individuals in the same age group. The slow-moving ganglioside was found to consist of glucose, galactose, and sialic acid in a molar ratio of 2:1:4, with no N-acetyl galactosamine detected (Ogiso et al. 1990). The composition of the slow-moving ganglioside was similar to GM3 except for the long-chain fatty acid moiety. Analysis by TLC further showed that an increase in gangliosides during cataract maturation was frequently accompanied by the appearance of polysialogangliosides (Ogiso et al. 1990).

We observed age-related changes in the distribution of the ganglioside-like antigen recognized by MAb 10A5. In 20-day-old animals, the antigen was found only in the 25/45 ocular lens fraction, but by age 75 days, the antigen was found in both the 25/45 fraction and NSMF. The redistribution of the ganglioside-like antigen from the major membrane fraction to both the major membrane fraction and the NSMF may be related to the aging-associated remodeling of the lens plasma membrane cytoskeleton (Beebe et al. 2001; De Maria et al. 2009; Lee et al. 2000). Redistribution of the antigen may have functional consequences. Gangliosides have been reported to modulate receptor function in microdomains (McJarrow et al. 2009), an effect that might be shared by the ganglioside-like antigen upon its incorporation into the NSMF. Furthermore, if the ganglioside-like antigen undergoes age-related modifications, it has the potential to contribute to cataractogenesis. Ogiso (1998) suggested that age-related modifications to lens gangliosides alter the cell-to-cell interaction induced by cell surface saccharide chains, resulting in initiation and progression of cataractogenesis. Modified gangliosides caused by age progression may also disrupt plasma membrane ion transport and trans-membrane signaling, further promoting age-dependent cataract formation (Hakomori 1981). Whether the ganglioside-like antigen modulates the function of the NSMF or contributes to cataract formation will require testing in an appropriate animal model of cataract.

## Conclusion

Using hybridoma technology, we showed that the 25/45 membrane fraction isolated from the ocular lenses of 20-day-old rats is antigenically distinct from the NSMF. Restriction of the antigen to the 25/45 fraction in 20-day-old animals supports the hypothesis that the 25/45 fraction and NSMF represent different subdomains within the ocular lens. MAb 10A5, a monoclonal antibody specific for this ganglioside-like antigen, will be a useful tool for tracking the antigen in an animal model of ocular aging and cataractogenesis.

## Methods

### Rat lens membrane preparations

All protocols involving animals were approved by the A.T. Still University Institutional Animal Care Committee and were conducted in accordance with the recommendations of the Guide for the Care and Use of Laboratory Animals. The 25/45 fraction and NSMF were isolated from the lenses of 20-day-old and 75-day-old Sprague Dawley rats as previously described (Fleschner 1998). Briefly, a 10% homogenate (wet tissue weight to buffer volume) was prepared by Dounce homogenization of decapsulated lenses in buffer comprising 5 mM Tris, 1 mM EDTA, and 5 mM β-mercaptoethanol, pH 8.0, containing a protease inhibitor cocktail (P8340) (Sigma Chemical Co., St. Louis, MO). The homogenate was centrifuged at 20,000 *g* for 30 min to obtain the water-insoluble sedimenting membrane fraction and water-soluble supernatant fraction (WSF). The sedimenting membrane fraction was further fractionated by discontinuous sucrose density gradient centrifugation through 25%, 45% and 50% sucrose at 100,000 *g* for 120 min. The 25/45 fraction was collected from the interface between 25% and 45% sucrose. The NSMF was isolated by adjusting the density of the water-soluble supernatant to 1.22 g/ml with solid KBr, centrifuging the solution at 100,000 *g* for 16 h, and removing the floating NSMF from the top of the solution. The NSMF was washed twice, dialyzed to reduce KBr concentration, and then concentrated by centrifugation at 68,000 *g* for 60 min.

### Hybridoma production and screening

Groups of four female BALB/c mice were immunized with the 25/45 fraction or NSMF isolated from 20-day-old rats in three intraperitoneal injections over the course of three months. Each injection contained 100 μg of protein in a total volume of 200 μl. For the first immunization, the antigen was emulsified in 50% Freund’s complete adjuvant (MP Biomedicals, Solon, OH). The second and third intraperitoneal injections were given in 50% Freund’s incomplete adjuvant (MP Biomedicals). Titers of sera obtained by tail bleed 11 days after the third intraperitoneal injection were determined by immunoblotting (Towbin et al. 1979) against the homologous antigen used for immunization. Three days prior to hybridoma production, the mouse from each group that had the highest antibody titer received an intravenous booster immunization containing 50 μg of antigen in 50 μl of phosphate-buffered saline (PBS, pH 7.4). Splenocytes from the mice were fused to Sp2/0-Ag14 myeloma cells as described by Van Deusen (1983) and selected in HAT medium prepared from Dulbecco’s modified Eagle’s medium supplemented with 15% horse serum (Sigma-Aldrich, St. Louis, MO).

Beginning 10 days after fusion, undiluted hybridoma culture supernates were screened for monoclonal antibodies (MAbs) by indirect enzyme-linked immunosorbent assay (ELISA) (Voller et al. 1976) against microtiter plates coated with 1 μg/well of 25/45 fraction or NSMF from 20-day-old rats. Reactive supernates were detected with 1:2000 alkaline phosphatase-conjugated goat anti-mouse immunoglobulins (IgG, IgM, IgA) (Sigma) and *p*-nitro phenyl phosphate substrate solution (Pierce Chemical Co., Rockford, IL). Supernatants that were reactive with the membrane fraction used for mouse immunization were subsequently tested by ELISA against the heterologous membrane fraction. From this screening protocol, a single monoclonal antibody was identified that reacted with one membrane fraction but not both: MAb 10A5 recognized an antigen unique to the 25/45 fraction that was absent from the NSMF in 20-day-old animals. The hybridoma line secreting MAb 10A5 was cloned three times by limiting dilution (Campbell 1984) and adapted to growth in HybriMax serum- and protein-free medium (Sigma). The MAb was concentrated and the medium was exchanged for PBS by ultrafiltration through Biomax-30 membranes (Millipore, Billerica, MA). MAb 10A5 was identified as an IgG2b antibody using a Mouse Typer™ Isotyping Kit (Bio-Rad, Hercules, CA).

### SDS-PAGE and immunoblotting of lens membrane fractions

Ocular lens fractions were subjected to SDS-PAGE as described by Laemmli (1970) through 18% or 5-20% polyacrylamide gradient resolving gels, and the proteins were visualized by staining with 0.1% w/v Coomassie Blue R-250 in 40% methanol/10% acetic acid. Alternatively, proteins were electrophoretically transferred from the gels to polyvinylidene fluoride (PVDF) membranes and probed by immunoblotting as described by Towbin et al. (1979). Briefly, membranes were blocked by a 1 h incubation in Tris-buffered saline (TBS; 20 mM Tris, 150 mM NaCl, pH 7.5) containing 5% nonfat dry milk (NFDM) before being incubated for 90 min in MAb 10A5 diluted to 1 μg/ml in TBS containing 0.05% Tween 20 (TTBS) and 1% NFDM. The membrane was washed in TTBS and then incubated for 90 min in 1:3000 alkaline phosphatase-conjugated goat anti-mouse IgG (Sigma). After further washes, membranes were developed with nitroblue tetrazolium/5-bromo-4-chloro-3-indolyl phosphate substrate (NBT/BCIP) (Bio-Rad).

### Enzymatic deglycosylation of the 25/45 fraction

The 25/45 fraction from 20-day-old rats was subjected to enzymatic deglycosylation (GlycoPro™ kit, Prozyme, San Leandro, CA) to remove N-linked (asparagine-linked) and simple O-linked (serine/threonine-linked) carbohydrates from glycoproteins. One hundred μg of 25/45 fraction protein in 30 μl of distilled water were mixed with 10 μl of 5X incubation buffer (0.25 M sodium phosphate, pH 7.0) and 2.5 μl of denaturation buffer (2% SDS, 1% 2-mercaptoethanol). The mixture was heated at 100°C for 5 min. After the mixture had cooled to room temperature, 2.5 μl of 15% NP-40 detergent were added, followed by 1 μl each of N-glycanase, sialidase A, and O-glycanase. Incubation with the enzymes was allowed to proceed for 3 h at 37°C, after which aliquots containing 8 μg of the deglycosylated 25/45 fraction protein were resolved by SDS-PAGE through 5-20% gradient gels. Control lanes were loaded with untreated 25/45 fraction protein (8 μg), and with untreated or deglycosylated bovine fetuin (1 μg/lane). The proteins were transferred to PVDF membranes for staining with Coomassie Blue R-250 or immunoblotting with MAb 10A5 as described previously. The membranes were examined to determine whether deglycosylation of the samples caused a shift in molecular weight or a reduction in reactivity with MAb 10A5 compared to untreated controls.

### Immunoprecipitation of the antigen reactive with MAb 10A5

Immunoprecipitation was performed as described by Stuart and Chamberlain (2003). An aliquot containing 150 μg of 25/45 fraction protein from 20-day-old rats was adjusted to a final concentration of 1% Triton X-100 (TX100) in a total volume of 150 μl PBS. The solution was sonicated on ice by three 15-sec bursts, incubated for 60 min in a 37°C water bath with occasional vortexing, and centrifuged for 10 min at 10,000 rpm in a minifuge. The supernatant was incubated for 90 min with 150 μl of Protein G Dynabeads (Invitrogen Corp., Carlsbad, CA) pre-coated with MAb 10A5 (1 μg antibody/μl beads). The beads were washed five times in PBS-0.01% Tween 20, and the immune complexes were eluted into 25 μl of Laemmli denaturing sample buffer (62.5 mM Tris–HCl, pH 6.8, 2% SDS, 5% 2-ME, 20% glycerol, 0.1% bromophenol blue) by incubation for 5 min in a boiling water bath. Eluates containing the immune complexes (12 μl/lane) were subjected to electrophoresis through 18% polyacrylamide gels. The gels were stained with Coomassie Blue R-250 as previously described, or with one of six alternative stains: colloidal Coomassie Blue G-250 (University of Missouri Proteomics Center 2006), Alcian blue (Wall and Gyi 1988), silver nitrate (Terry et al. 2004), imidazole-zinc sulfate (Castellanos-Serra et al. 1999; Hardy et al. 1997), Stains-All (Goldberg and Warner 1997), or a combination of Stains-All and silver nitrate (Goldberg and Warner 1997; Terry et al. 2004). The colloidal Coomassie Blue G-250 staining procedure was used to fix and stain gels in preparation for MALDI-ToF/MS, described below. The gel was washed three times in distilled water, stained overnight at room temperature in 300 ml of Coomassie Brilliant Blue G-250 (0.08% w/v in 20% ethanol, 1.6% phosphoric acid, and 8% ammonium sulfate), and then destained by extensive washing in distilled water.

### 2-Dimensional (2-D) electrophoresis

The antigen reactive with MAb 10A5 was immunoprecipitated from 450 μg of TX100-solubilized 25/45 fraction using 270 μl of MAb 10A5-coated Protein G Dynabeads. Immune complexes were eluted from the beads into 375 μl of isoelectric focusing (IEF) sample buffer (9 M urea, 4% CHAPS, 3 mM tributylphosphine, 0.04% Bio-Lyte 3/10 ampholytes) by incubation for 1 h in a 37°C water bath with occasional vortexing. The eluate was split into three 125-μl portions, each of which was used to rehydrate a 7-cm ReadyStrip™ IPG strip (Bio-Rad), pI 3–10, under active rehydration conditions. The strips were focused in a programmable Protean IEF cell (Bio-Rad), after which they were equilibrated by successive 10-min incubations in buffer I (6 M urea, 0.375 M Tris–HCl, pH 8.8, 2% SDS, 20% glycerol, 2.2% dithiothreitol) and buffer II (6 M urea, 0.375 M Tris–HCl, pH 8.8, 2% SDS, 20% glycerol, 1.25% [w/v] iodoacetamide). The IPG strips were then subjected to second-dimension SDS-PAGE through 18% polyacrylamide gels. The contents of one gel were transferred to a PVDF membrane and immunblotted with MAb 10A5 to confirm the presence of the antigen. The two replicate gels were fixed and stained with colloidal Coomassie Blue G-250.

### MALDI-ToF and MS analysis

For MALDI-ToF/MS, gel slices containing the antigen immunoprecipitated by MAb 10A5 were excised from 1-D and 2-D gels after fixation and staining in colloidal Coomassie Blue G-250. Because the antigen did not take up the dye, it was localized in the gels by comparison to replicate immunoblots probed with MAb 10A5. As an added assurance that gel slices from 2-D gels contained the immunoreactive antigen, ends of the excised pieces were probed with MAb 10A5 by immunoblotting. A 1-mm piece clipped from both ends of each slice were equilibrated in denaturing sample buffer, subjected to SDS-PAGE, transferred to PVDF, and probed with MAb 10A5. Gel slices shown to contain the antigen were submitted to Applied Biomics, Inc. (Hayward, CA) for trypsin digestion and MALDI-ToF/MS on an Applied Biosystems Proteomics analyzer. Mass lists compiled from the mass spectra were searched against the National Center for Biotechnology Information non-redundant (NCBInr) mammalian protein database using GPS Explorer software equipped with the MASCOT search engine.

### Protease digestion of the 25/45 fraction

The 25/45 fraction from 20-day-old rats was subjected to in-solution trypsin digestion as described in the product bulletin (Part# 9PIV511) for TPCK-modified sequencing grade trypsin (Promega, Madison, WI). A sample containing 100 μg of 25/45 fraction protein dissolved in 50 μl of protein denaturation buffer (50 mM Tris–HCl, 6 M urea, 4 mM DTT, pH 8.0) was digested at 37°C with 5 μg of trypsin in 300 μl of digestion buffer (50 mM Tris–HCl, 1 mM CaCl_2_, pH 7.6). Aliquots containing 20 μg of the 25/45 fraction were removed after 0 h (undigested control containing no enzyme), 2 h, 24 h, and 48 h of incubation. Digestion was stopped by adding 3 μl of 50 mM phenylmethylsulfonyl fluoride (PMSF) to each aliquot and immediately storing the samples at -80°C. Digestion with proteinase K (Fisher Scientific, St. Louis, MO) was performed in similar fashion, except that proteinase K was used at a concentration of 0.1 μg/μl in digestion buffer comprising 50 mM Tris–HCl, 5 mM CaCl_2_, pH 7.5, and digestion was stopped by adding 5 μl of 50 mM PMSF to each 20 μg aliquot. All samples were lyophilized to dryness and then reconstituted in 30 μl of Laemmli sample buffer prior to SDS-PAGE through duplicate 18% resolving gels (15 μl/lane). Gels were stained with Coomassie Blue R-250 or immunoblotted with MAb 10A5 as previously described.

### Folch extraction of the 25/45 fraction and thin-layer chromatography

A sample containing 400 μg of 25/45 fraction protein from 20-day-old rats was subjected to the Folch lipid extraction procedure (Folch et al. 1957). The upper phase, interface, and lower phase were collected into separate glass tubes, lyophilized to dryness, and rehydrated in 60 μl of PBS. Fifteen μl from each sample were mixed with 5 μl of 4X Laemmli sample buffer and incubated in a boiling water bath for 5 min. Each 20-μl sample was then subjected to SDS-PAGE and immunoblotting to determine which fraction(s) contained the antigen recognized by MAb 10A5.

The upper phase obtained by Folch extraction from 200 μg of 25/45 fraction protein was analyzed by thin-layer chromatography (TLC) on duplicate 20 × 20 cm, 250 micron Uniplate™ Silica Gel H, binder-free plates (Analtech, Newark, DE) with a propanol/water (7:3 v/v) solvent system. One TLC plate was stained with resorcinol (Findlay and Evans 1990), while the duplicate plate was used to recover gangliosides for immunoblotting. For ganglioside recovery, consecutive 2 × 2 cm silica gel bands were scraped from lanes containing the upper phase and eluted into 1 ml of chloroform/methanol (1:1 v/v). Volumes were reduced under a nitrogen stream, and samples were solubilized in 40 μl of Laemmli sample buffer prior to SDS-PAGE (20 μl/lane) and immunoblotting with MAb 10A5.

The upper phase of the 25/45 fraction Folch extract was also subjected to high performance TLC on 10 × 10 cm HPTLC Silica Gel 60 plates (Fisher Scientific) (Schlosshauer et al. 1988). Gangliosides extracted from the equivalent of 20, 9.7, and 6.4 μg of 25/45 fraction protein were spotted onto the HPTLC plates in 1 μl of chloroform/methanol (2:1 v/v) along with bovine mixed ganglioside standards (Matreya, Pleasant Gap, PA) in adjacent lanes. Additional samples applied to the HPTLC Silica Gel 60 plates included the proteoglycans chondroitin sulfate and heparan sulfate, each applied at 10 μg. Plates were developed in a solvent system comprising chloroform/methanol/0.2% aqueous CaCl_2_ (55:45:10 v/v/v) and stained with resorcinol.

### Ganglioside dot blot immunoassay

MAb 10A5 was tested for reactivity against bovine gangliosides by a dot blot immunoassay adapted from the method of Chabraoui et al. (1993). A PVDF membrane was wetted in methanol, soaked for 5 min in PBS, and then inserted while still wet into a dot blot apparatus (Bio-Rad). Membrane spots were dried by vacuum pressure and then coated with antigens solubilized in methanol (1.5 μl/spot). Antigens included bovine mixed gangliosides (5 μg), purified bovine GD1a (1 μg, Matreya), the Folch upper phase extracted from 2 μg of 25/45 fraction protein, and 50 ng of the irrelevant bacterial protein RadA-6xHis (Richardson et al. 2012), the latter serving as a negative control. After a 90-min incubation at room temperature to facilitate antigen adsorption, the membrane was removed from the apparatus, rinsed in PBS, and blocked for 60 min in PBS containing 5% bovine serum albumin (BSA). Replicate strips cut from the membrane were incubated for 60 min in MAb 10A5 or an isotype-matched negative control antibody [MAb 2A2 specific for RadA-6×His (Richardson et al. 2012)], each diluted to 2 μg/ml in PBS-1% BSA. After several washes in PBS, the strips were incubated for 60 min in 1:2000 goat anti-mouse IgG-alkaline phosphatase, washed again, and then developed in NBT/BCIP substrate. To demonstrate that gangliosides remained bound to the PVDF membrane throughout the dot blot protocol, one of the replicate strips was stained with Coomassie Blue R-250 to enable visualization of white ganglioside spots against a dark blue background.

## Electronic supplementary material

Additional file 1: **Peptide summary report for 1-D gel slice containing the antigen recognized by MAb 10A5.** (XLS 58 KB)

Additional file 2: **Peptide summary report for two 2-D gel slices containing the antigen recognized by MAb 10A5.** (XLS 739 KB)
